# Supplementary material for: Netcombin: An algorithm for constructing optimal phylogenetic network from rooted triplets
Source: PLoS One. 2020 Sep 18;15(9):e0227842. doi: 10.1371/journal.pone.0227842 (PMC7500971; doi:10.1371/journal.pone.0227842)
Supplement: S4 File — (ZIP) [file pone.0227842.s004.zip › SequenceData-Triplet/sequenceData-10-20-40-Triplet2/data-(sample 10- length 500- num seq 40- m rate 10^-6)/7 seq.pdf]

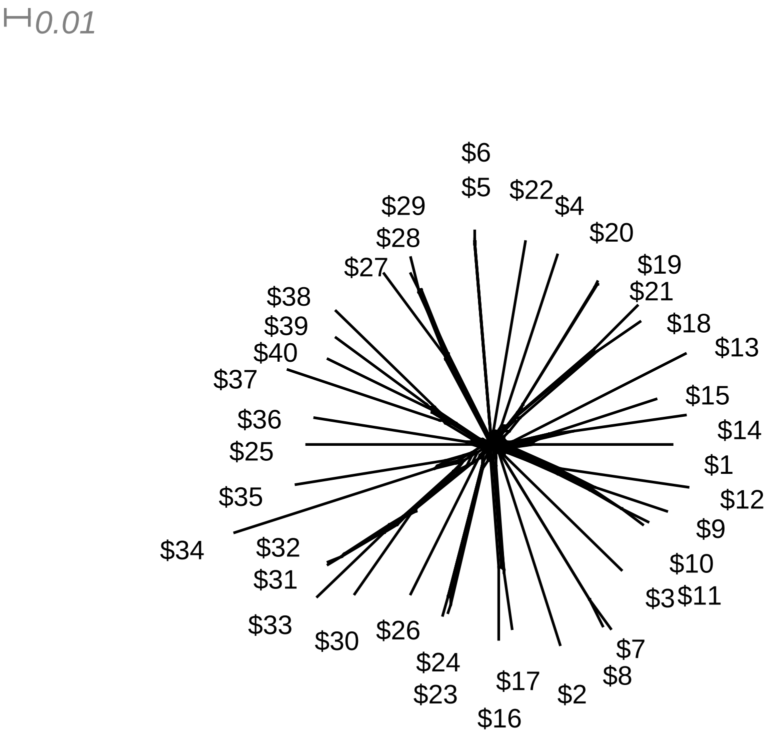

0.01
$6
$5
$22
$4
$20
$19
$21
$18
$13
$15
$14
$1
$12
$9
$10
$3
$11
$7
$8
$2
$17
$16
$23
$24
$26
$30
$33
$31
$32
$34
$35
$25
$36
$37
$40
$39
$38
$27
$28
$29
